# Double pituitary adenoma associated with acromegaly and hyperprolactinemia: a case report

**DOI:** 10.20945/2359-4292-2024-0065

**Published:** 2025-04-03

**Authors:** María Gabriela García Falcone, Santiago Gonzalez Abbati, Soledad Sosa, Andrea Paes de Lima, Florencia Peralta, Karina Danilowicz

**Affiliations:** 1 División Endocrinología, Hospital de Clínicas José de San Martín, Universidad de Buenos Aires, Buenos Aires, Argentina; 2 Departamento de Neurocirugía, Hospital de Clínicas José de San Martín, Universidad de Buenos Aires, Buenos Aires, Argentina; 3 Departamento de Patología, Hospital de Clínicas José de San Martín, Universidad de Buenos Aires, Buenos Aires, Argentina

## Abstract

Doubleor multiple adenomas are rare, and synchronous secretory pituitary adenomas are rarer still. We report a case of a 30-year-old woman with a 6-year history of amenorrhea and occasional galactorrhoea. She presented with headaches, weight gain, subtle acromegalic features, new-onset hypertension and diabetes. Workup confirmed acromegaly and hyperprolactinemia. Preoperative magnetic resonance imaging of the pituitary demonstrated two noncontiguous microadenomas. Two distinct tumors were resected through a transsphenoidal approach. Immunohistochemical analysis of each separated adenoma confirmed the diagnosis of acromegaly and prolactinoma. Postoperatively, she was cured of acromegaly, and her amenorrhea/galactorrhea syndrome resolved. Her growth hormone and insulin-like growth factor-I levels normalized, whereas her prolactin level remained slightly above normal. Therefore, it is critical to consider double or multiple adenomas preoperatively through careful endocrine assessment and review of magnetic resonance imaging. As shown in our case, careful evaluation led to a better surgical outcome.

## INTRODUCTION

Pituitary adenomas or pituitary neuroendocrine tumors (PitNETs), according to the newest nomenclature coined in the 2021 5^th^ edition World Health Organization (WHO) Classification of Central Nervous System Tumors (CNS5) and 2022 5^th^ edition WHO Classification of Endocrine and Neuroendocrine Tumors (ENDO5), are present in up to 20% of the population (^1^), accounting for 10 to 15% of all brain tumors. This updated nomenclature was introduced to unify pituitary adenomas with other neuroendocrine neoplasias. However, concerns and mixed acceptance among different pituitary research societies have arisen, suggesting that it does not reflect prognosis and may adversely affect patient care (^2,3^).

Synchronous multiple PitNETs are defined as unique tumors separated clearly by nontumorous tissue (^4-6^), although some authors have considered contiguous tumors (^7^) or even more than one tumor type of different cell lineages in a single lesion (^8^).

Multiple PitNETs are rare, with an incidence of 1 to 9% at autopsy and 0.2 to 2.6% in surgical series (^4,9,10^). Acromegaly and Cushing’s disease are the most common presentations of coexisting PitNETs (^4,8,10^). Functioning PitNETs often also coexist with nonfunctioning tumors (^8^), most commonly silent lactotrophs, silent corticotrophs, silent somatotrophs and gonadotroph adenomas (^4,11^). However, there are reports of patients presenting with two distinct hypersecretory syndromes, predominantly Cushing’s disease associated with hyperprolactinemia and acromegaly associated with hyperprolactinemia (^6,7^). In a recent systematic review by Zhang et al. including 59 patients with double PitNETs, the most prevalent clinical manifestation was Cushing’s disease (39%), followed by acromegaly (34%), and combined endocrine symptoms were uncommon, with manifestations of acromegaly and hyperprolactinemia symptoms in 5% of the patients (^12^).

Herein, we report a case of a young woman with a double adenoma and provide additional information on this condition.

## CASE REPORT

We report the case of a 30-year-old woman who was referred to our institution with a 6-year history of amenorrhea.

She had been evaluated 6 years before for amenorrhea and infertility and was found to have hyperprolactinemia. She received treatment with cabergoline and achieved spontaneous menstruation and a successful pregnancy, so cabergoline was stopped. Pregnancy and delivery had no complications. Thereafter, spontaneous menstruation did not restart. She reported occasional galactorrhoea.

On anamnesis, she reported a 1-year history of headaches, swelling of hands and feet, arthralgia and an increase in shoe and ring size. She had gained 15 kg in the last 3 years. Her blood pressure was elevated and controlled with an antihypertensive drug, and she was recently diagnosed with diabetes.

Her family history was unremarkable, with no known familial pituitary adenomas or multiple endocrine neoplasia (MEN) syndrome.

The data collected from her physical examination included height (165 cm), weight (108 kg), body mass index (39 kg/m^2^), blood pressure (120/80 Hgmm), and heart rate (68 bpm). She had central fat distribution without abdominal striae. She had subtle facial acromegalic features, including prognathism, prominent nose bridge and thickened lips. Ophthalmologic examination with visual field testing and neck examination with thyroid gland assessment were unremarkable.

Her outpatient workup revealed hyperprolactinemia, elevated basal serum levels of insulin-like growth factor (IGF-I), hypogonadotropic hypogonadism, subclinical primary hypothyroidism with autoimmune thyroid antibodies and normal serum calcium **(Table 1)**.

**Table 1:** Preoperative and follow-up hormonal evaluation

	Preoperative	3 months postoperative	8 months postoperative	12 months postoperative	41 months postoperative
IGF1 ng/mL (149-247)	740	115	122	102	91
GH ng/mL (0-10)	3.77	0.05	0.06	0.47	0.9
PRL ng/mL (4.73-23.3)	222.8	31.2	22	49	32
Cortisol mcg/dL (5.2-19.4)	8	3.4	4.2	4.3	5.8
FSH mIU/mL (4.7-21.5)	2.88			3.9	
LH mIU/mL (5-25)	1.41			2.2	
Estradiol pg/mL	< 16			16.6	
TSH UI/mL (0.27-4)	6.8	0.88	3.2	7.35	3.2
Free T4 ng/dL (0.9-1.7)	1.05	1.16	1.06	0.8	1.06
ATPO (< 34)	373				390
ATG (< 115)	89				115
Serum calcium mg/dL (< 10)	9.4			9.1	

IGF: insulin-like growth factor; GH: growth hormone, PRL: prolactin, FSH: follicle-stimulating hormone; LH: luteinizing hormone; TSH: thyroid-stimulating hormone; ATPO: anti-thyroid peroxidase antibodies, ATG: anti-thyroglobulin antibodies.

**Figure 1:**
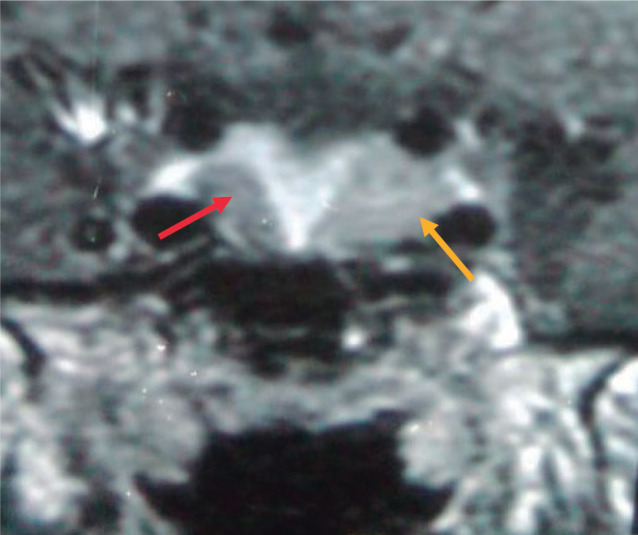
Magnetic resonance imaging demonstrating two distinct pituitary adenomas. Coronal magnetic resonance image with gadolinium contrast enhancement. The red arrow indicates the right-sided tumor, and the yellow arrow indicates the left-sided tumor.

Subsequent magnetic resonance imaging (MRI) of the pituitary gland revealed two clearly separate adenomas. A hypointense lesion in the left half of the sella was consistent with a pituitary adenoma measuring 5 mm × 10 mm (Knosp grade II), and a similar lesion in the right half of the sella measuring 4 mm × 8 mm (Knosp grade I) was detected **(Figure 1)**. The pituitary stalk deviated to the right side.

Medical treatment with a dopamine agonist was proposed, but she preferred the surgical option. The patient underwent exploratory pituitary surgery through the transsphenoidal approach. Two distinct tumors were identified and resected as two separate specimens.

**Figure 2:**
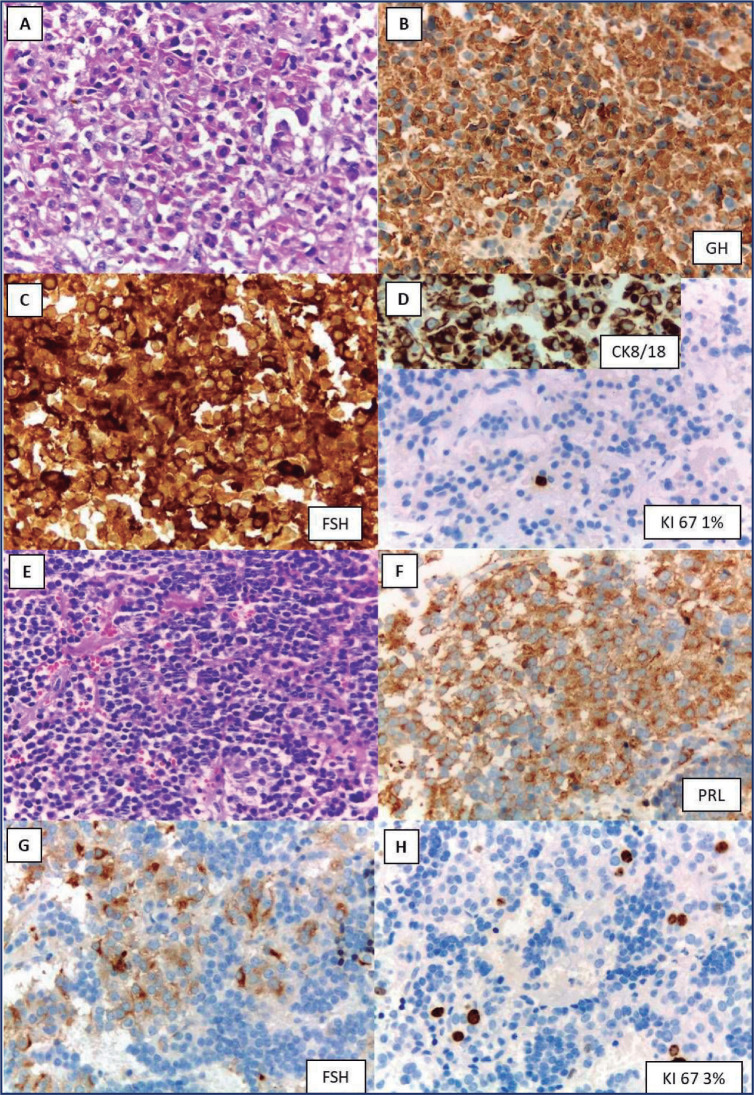
Histopathology of right (**A:** hematoxylin and eosin; **B:** GH; **C:** follicle-stimulating hormone; **D:** CK8/18 perinuclear and cytoplasmic; Ki67 1%) and left (**E:** hematoxylin and eosin; **F:** PRL; **G:** follicle-stimulating hormone; **H:** Ki67 3%) pituitary neuroendocrine tumors (250x).

Histologic examination revealed two pituitary microadenomas. Immunohistochemistry revealed that the left adenoma was positive for PRL and follicle-stimulating hormone (FSH) and negative for all other pituitary hormones. Ki67 was 3%. The right adenoma was positive for GH and FSH. Histology was compatible with a densely granulated somatotroph tumor. Ki67 was 1% **(Figure 2)**.

FSH: follicle-stimulating hormone, GH: growth hormone, PRL: prolactin.

The patient had an uneventful postoperative course. Treatment with levothyroxine for subclinical primary hypothyroidism was started before surgery, and hydrocortisone was replaced postoperatively.

Three months later, she experienced marked symptomatic improvement and resumption of spontaneous menses. Her hypertension and diabetes mellitus resolved, and she lost 10 kg of weight.

Her cortisol level remained low postoperatively; hence, she was still on hydrocortisone replacement 60 months after surgery.

Growth hormone and IGF-I serum levels normalized. The GH level was < 0.1 ng/mL at 3 months after surgery, and the IGF-I values remained significantly below the normal levels adjusted for age at 18 months postsurgery. On the other hand, the serum prolactin level remained slightly abnormal at 3 and 18 months postoperatively.

Postoperative sellar MRI 60 months after surgery revealed no residual tumor tissue.

## DISCUSSION

Multiple PitNETs are rare entities, and the majority of PitNETs have been documented in case reports and surgical or autopsy series.

Pituitary adenomas are thought to be monoclonal on the basis of X chromosome inactivation patterns (^13^). Many germline genetic variants are responsible for pituitary adenomas in the familial context (*MEN1, PRKAR1A, AIP, CDKN1B, GPR101, SDHA, SDHB, SDHC, SDHD*, and *SDHAF2*), accounting for 5% of them (^14^). *GNAS* and *USP8* are somatic variants associated with sporadic pituitary tumorigenesis, and epigenetic modifications affecting gene expression, including DNA methylation, histone modification and ARN interference, are increasingly being recognized (^15^).

The pathogenesis of double pituitary adenomas is not known, but different possible mechanisms may be considered: the occurrence of two monoclonal expansions of anterior pituitary cells, the occurrence of different clonal proliferations within one original adenoma or the induction of the genesis of the second lesion due to the production of growth factors from the original adenoma (^5^). A possible genetic background should also be considered since some of the reported cases of double pituitary adenomas arise clinically from MEN (^4^).

We presented a patient with a double pituitary adenoma comprising two functional adenomas: a somatotropinoma and a microprolactinoma. The main clinical finding was related to hyperprolactinemia first, followed by acromegalic signs several years later.

According to the 2017 WHO classification of endocrine tumors, multiple tumors must originate from different cell linages to be considered multiple PitNETs (^16^), but can be considered to be multiple PitNETs if both tumors coexist and are separate (^9^).

In addition to immunohistochemical evidence of differential hormone expression, nuclear transcription factor detection and specific DNA methylation profiling analyzed using established algorithms are useful for identifying lesions as different adenomas occurring in the same patient (^17^). Transcription factors lead to cell linage differentiation from pituitary stem cells: pituitary-specific POU-class homeodomain transcription factor (PIT-1) is expressed in GH-PRL-TSH cells, steroidogenic Factor 1 (SF1) drives gonadotroph cells, and the T-box family member TBX19 (T-PIT) directs corticotroph cells (^18^). Unfortunately, these DNA methylation and transcription factor based techniques are not broadly available in Argentina, limiting the precise diagnosis of separated adenomas when the microscopic picture is not definitive.

Regarding PitNET types in double adenoma series, the German group recently reported sparsely granulated prolactin cells as the most common tumor type (^9^), although other authors described GH-secreting PitNETs as the most common type (^8^), and both were present in our patient.

Generally, in the setting of simultaneous excessive GH and prolactin secretion, the two hormones are most often considered to be secreted by the same adenoma or hyperprolactinemia due to stalk compromise. However, there are few reports of synchronous GH- and prolactin-secreting adenomas (^19-21^), some of which are even associated with familial MEN type 1 (^21^). Thus, it cannot be assumed that the two hormones are secreted by the same adenoma, and a very careful preoperative review via MRI must be carried out to identify the possible presence of two distinct adenomas, as in our case.

When double adenomas are present, it is critical to ensure that surgery removes both adenomas. A lack of awareness of multiple pituitary adenomas and nondiagnosis on available preoperative radiologic images are risk factors for surgical failure (^22^). In this case, we suggested cabergoline as a first-line treatment; however, the patient preferred surgery. This preference has become widespread as a first-line therapeutic indication with 71 to 100% efficacy (achieving normoprolactinemia), considering the favorable cost-benefit analysis over medical treatment, especially in young patients (^23,24^). The recognition of double pituitary adenomas prior to surgery is essential for providing a curative treatment plan.

To the best of our knowledge, few data are available regarding evolution: Ogando-Rivas et al. reported the need for reoperation in 2 out of 17 cases of double adenomas (one immediately postoperatively due to tumor persistence and the other 14 months after surgery because of tumor regrowth) (^6^). Budan et al. reported structural persistence/recurrence in 3 out of 63 patients (whose tumors presented as PRL-GH, GH-ACTH and ACTH-GH) and biochemical persistence in 4 of them (^4^). Zieliński et al. reported biochemical persistence in 2 out of 22 cases of double adenomas (^10^). Rahman et al. reported a case of synchronous GH and prolactin-secreting pituitary adenomas with persistent hyperprolactinemia after surgery (^19^). Similarly, our patient was cured after surgical treatment for acromegaly, and her amenorrhea/galactorrhea syndrome resolved, although her prolactin levels remained slightly elevated.

## CONCLUSIONS

Double or multiple adenomas are rare but should be considered preoperatively. High suspicion when evaluating magnetic resonance imaging is of utmost importance. This must be accompanied by a thorough preoperative endocrine assessment. As shown in our case, this careful evaluation led to optimal surgical outcomes.
